# Changes in mast cell infiltration: a possible mechanism in detrusor overactivity induced by visceral hypersensitivity

**DOI:** 10.1590/S1677-5538.IBJU.2015.0025

**Published:** 2016

**Authors:** Nian-zhao Zhang, Lin Ma, Chen Jun, Yan-xia Guo, Hui-qing Yuan

**Affiliations:** 1 Department of Urology, Qilu Hospital, Shandong University, Jinan, P.R. China; 2 Department of Biochemistry and Molecular Biology, Shandong University School of Medicine, Shandong University, Jinan, P.R. China

**Keywords:** Urinary Bladder, Overactive, Hypersensitivity, Mast-Cell Sarcoma

## Abstract

**Objective:**

To establish the detrusor overactivity (DO) model induced by visceral hypersensitivity (VH) and investigate the relationship between mast cell (MC) infiltration and DO.

**Materials and Methods:**

Sixty rats are divided into 4 groups randomly: Group 1:Baseline group; Group 2: DO group; Group 3: CON group; Group 4: VH group. The colorectal distension (CRD) and abdominal withdral reflex (AWR) scores are performed to evaluate VH. The cystometric investigation and histological test of MC infiltration are assessed.

**Results:**

The threshold pressure of CRD in the VH group is significantly lower than that in the CON group (P<0.001). At the distension pressure ≥20 mmHg, the AWR scores of the VH group are significantly higher than those of the CON group (10 mmHg: P=0.33; 20 mmHg: P=0.028; 40 mmHg: P<0.001; 60 mmHg: P<0.001; 80 mmHg: P<0.001). DO model is successfully established in the VH group (DO rate=100%). Compared with the CON group, the numbers of MC infiltration are significantly increased in the VH group, including submucosa of bladder (P<0.001), mucosa lamina propria/mesentery of small intestine (P<0.001), and mucosa lamina propria/mesentery of large intestine (P<0.001). Furthermore, more MC activation as well as degranulation are observed in the VH group.

**Conclusions:**

It is indicated that DO model can be established in the VH rats. The MC infiltration may play an important role in DO induced by VH, and may be helpful to understand the mechanisms of DO in VH patients.

## INTRODUCTION

Overactive bladder syndrome (OAB) is a symptom complex consisting of urinary urgency, usually accompanied by frequency and nocturia, with or without urinary incontinence. OAB is usually associated with involuntary contractions of the detrusor muscle, which can result in urge incontinence, depending on the response of the sphincter. The most common cause of OAB is detrusor overactivity (DO) (1, 2). DO is defined as the occurrence of involuntary detrusor contractions during filling cystometry. This diagnosis by symptoms and urodynamic investigations is made in patients with lower urinary tract symptoms when involuntary detrusor muscle contractions occur during filling cystometry (1, 3). Sixty-four percent of all patients with OAB have DO on cystometry, and 69% of men and 44% of women with urgency have DO (4). DO may occur in male patients with bladder outlet obstruction, which leads to several structural and functional changes in the detrusor muscle. On the other hand, DO is thought to result not only from efferent (motor) hyperfunction/dysfunction but also most likely by afferent (sensory) noise. However, the pathogenesis of DO currently is still unclear.

Visceral hypersensitivity (VH), a consistent finding in a large proportion of patients with irritable bowel syndrome (IBS), is currently considered a key pathophysiological mechanism involved in pain perception in large subgroups of patients with functional gastrointestinal disorders (5, 6). Furthermore, mast cell (MC) activation is thought to be involved in VH, one of the main characteristics of IBS (7-10). Patients with IBS may have a disorder of smooth muscle or its innervation that is not confined to the gastrointestinal system (11, 12). Although IBS is marked by abdominal pain and alterations in bowel movement frequency or form, patients with this disorder may also experience urinary symptoms as DO, including frequency, urgency and dysuria (13). In a study of female patients, urinary urgency and frequency were significantly more common in those with IBS than in controls (14). The functional connection between the bladder and bowel, evidenced in studies of the neural crosstalk between these two organs, suggests that they may share some common underlying dysfunction (11, 15). However, the exact pathophysiology of DO in patients with IBS is not well-understood.

Although several epidemiological studies showed the association between the bladder and bowel (11, 12, 15), less experimental work has been performed to study the relationship between DO and VH in the animal model. It is important and interesting to investigate the relationship between DO and VH, and the role of MC in DO model induced by VH. In our study, we established the DO model induced by VH to achieve a better understanding of MC in DO.

## MATERIALS AND METHODS

### 

#### Animals

Adult female Wistar rats (200-250g), obtained from the Experimental Animal Center of Shandong University) were used in our study. The animals were housed under standardized conditions of temperature (20±1ºC), humidity (50±5%) and lighting (12-h day/12-h night). All experimental procedures were performed at the same time of the day between 9:00am and 12:00am to avoid the effect of diurnal variations. This study was approved by Chinese Institutional Animal Care Committee and adhered to the ethical guidelines of the International Association for the Study of Pain. Great care was taken to minimize or avoid discomfort to the animals.

#### Experimental treatments

Sixty rats were divided into 4 groups randomly: Group 1: no intervention for establishing baseline values (Baseline group); Group 2: DO (without VH) induced by obstruction according to previous study (DO group) [18]; Group 3: the control gave sodium chloride (CON group); Group 4: thirty milligrams chicken egg albumin (Sigma Co., USA, purity grade V) joined with 1mL aluminum hydroxide gel with the mass concentration of 10mg/mL (Sigma Co., USA), then mixed well before each rat accepted intraperitoneal injection according to previous study (VH group) (16). The colorectal distension (CRD) and abdominal withdrawal reflex (AWR) were performed to evaluate VH, then the filling cystometry was performed to investigate the possibility of DO, and the relationship between DO and VH was evaluated. Lastly a light microscope was used to observe the MC infiltration of the bladder and intestine tissue stained by toluidine blue.

#### Threshold pressure and AWR Scores (17)

CRD in rats induces the contractions of abdominal and hind limb musculature, which has been validated as a quantitative measure of VH. Behavioral responses to CRD were assessed by measuring the threshold pressure that induced the first abdominal contraction and the AWR with a scoring system.

Briefly, rats were lightly sedated with halothane while a flexible latex balloon (4cm) made of a surgical glove finger attached to a Tygon tubing with thread was inserted intra-anally into the descending colon and rectum until the thread end was 1cm proximal to the anal sphincter. And the balloon was secured in place by taping the tubing to the tail. Rats were then placed in small Lucite cubicles (20×8×8cm) and allowed 30 min for recovery from sedation before testing. For measuring the threshold pressure of CRD, the colorectal balloon was progressively inflated with an increment of 5mmHg until the pain behavior displayed or until a cutoff pressure of 80mmHg was reached in order to avoid invincible damage to the rat. For measuring the AWR, the balloon was rapidly inflated to constant pressure [10, 20, 40, 60, 80mmHg]. The AWR scores were graded on a scale of 0 to 4: 0: no behavioral response to CRD; 1: brief head movement followed by immobility; 2: contraction of abdominal muscles; 3: lifting of abdomen; 4: body arching and lifting of pelvic structures. For the both measurements (threshold pressure and AWR), the animals were given CRD with 30-s duration and then 4-min interval. All the measurements were assessed by two blinded observers in triplicate.

## URODYNAMIC AND HISTOLOGICAL TEST

### 

#### Urodynamic test

In the cystometric investigation, the conscious rats were held under partial restraint in a restraining device (18). The bladder was catheterized through urethra by a human epidural catheter (F2, 0.7-mm outer diameter, 0.4-mm internal diameter), which was connected via a T-tube to urodynamic testing machine (Laborie medical technologies, Corp) and infusion pump (LION WZ-50C6 microinfusion pump, Zhejiang University, China).

The rats were placed supine and the urethral orifice could be observed clearly. Data were collected and analyzed after the animals were seen to be resting quietly in the restraining device. The cystometric investigation was performed by infusing warm saline (37-38ºC) at a rate of 9mL/h, and the infusion was stopped when voiding contraction was observed according to the voiding pressure and the leakage of urine around the urethral orifice. Bladder emptying was ascertained by opening the catheter and gently pressing the lower abdomen after the cystometry. During the filling phase, some of the rats had obvious non-voiding contractions before the onset of voiding contraction and thus were defined as having DO (19). According to the previous study (18), three urodynamic cycles per animal were recorded to insure reproducibility of the bladder responses.

#### Histological test of MC

After urodynamic test and micturition for thirty minutes, each rat was successfully anesthetized (10% chloral hydrate, 3mL/kg, i.p.). In each rat, a median incision of the abdomen was chosen to expose the organs, and the bladder and intestine were quickly removed. Histological changes in the bladder and intestine tissue were observed by the stained by toluidine blue to research MC. The numbers of MC were counted in 5 consecutive fields (×400), and the mean values were used in the subsequent statistical analysis. The urodynamic and histological test were assessed by two blinded observers in triplicate.

### Statistical analysis

The model data were expressed as mean±standard deviation (SD), and SPSS 16.0 statistical software (SPSS Inc., IL, USA) was used to conduct the t test. P value <0.05 was considered statistically significant.

## RESULTS

### 

#### Threshold Pressure of CRD

**Figure 1:**
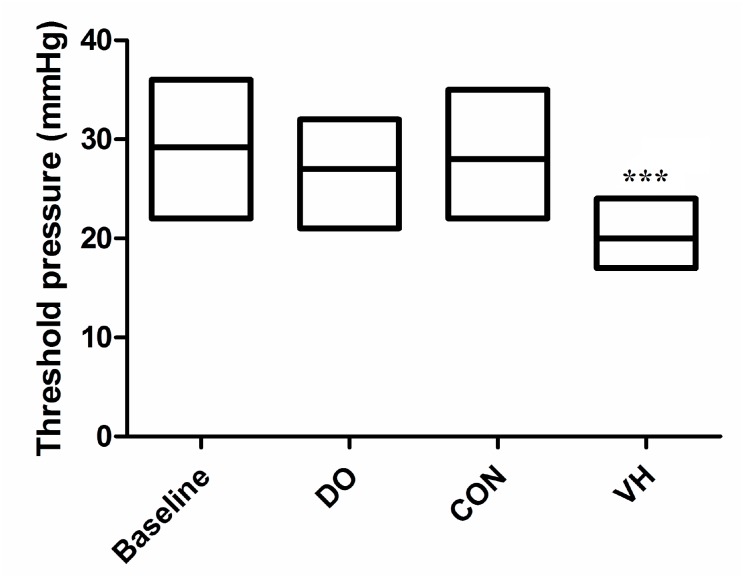
Comparison of threshold pressure in response to CRD between CON and VH group (29±6.1 versus 22±3.4, ***P<0.001).

The threshold pressure to elicit a distinctive abdominal muscle contraction in response to CRD was recorded in the 4 groups, respectively. Compared with the CON group, the visceral responses of the VH group to CRD were significantly greater. The results showed that the threshold pressure of CRD in the VH group was significantly lower than that in the CON group (22±3.4mmHg versus 29±6.1mmHg, P<0.001, Figure-1).

#### AWR Scores

**Figure 2:**
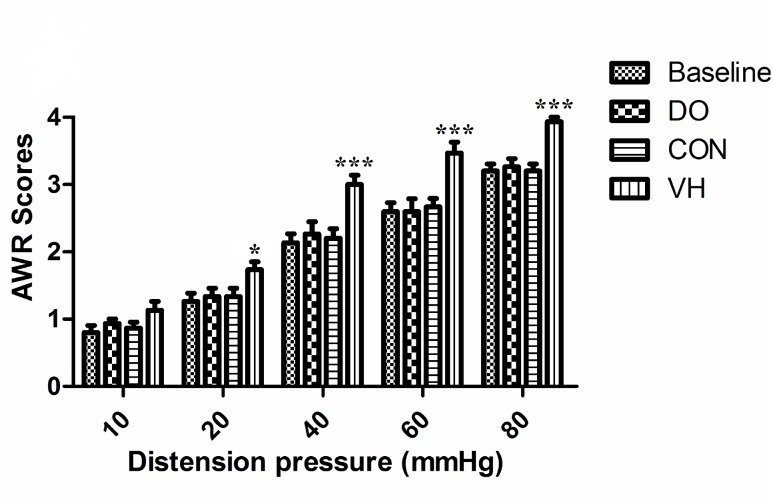
Comparison of AwR scores between CON and VH group.(10mmhg: 0.87±0.12 versus 1.0±0.13, P=0.33; 20mmhg: 1.3±0.22 versus 1.7±0.20, *P=0.028; 40mmhg: 2.2±0.29 versus 3.0±0.27, ***P <0.001; 60mmhg: 2.7±0.22 versus 3.5±0.38, ***P<0.001; 80mmhg: 3.2±0.16 versus 3.9±0.062, ***P<0.001).

A series of graded responses were shown in the 4 groups with the balloon pressure increasing from 10 to 80mmHg. At the distension pressure 10mmHg, the mean AWR scores in the VH group approximated to those in the CON group. At the distension pressure ≥20mmHg, the rats in the VH group exhibited stronger responses than those in the CON group as reflected by significantly higher AWR scores (10mmHg: 1.0±0.13 versus 0.87±0.12, P=0.33; 20 mmHg: 1.7±0.20 versus 1.3±0.22, P=0.028; 40mmHg: 3.0±0.27 versus 2.2±0.29, P<0.001; 60mmHg: 3.5±0.38 versus 2.7±0.22, P <0.001; 80mmHg: 3.9±0.062 versus 3.2±0.16, P <0.001; Figure-2).

#### Urodynamic and histological test of MC

**Figure 3:**
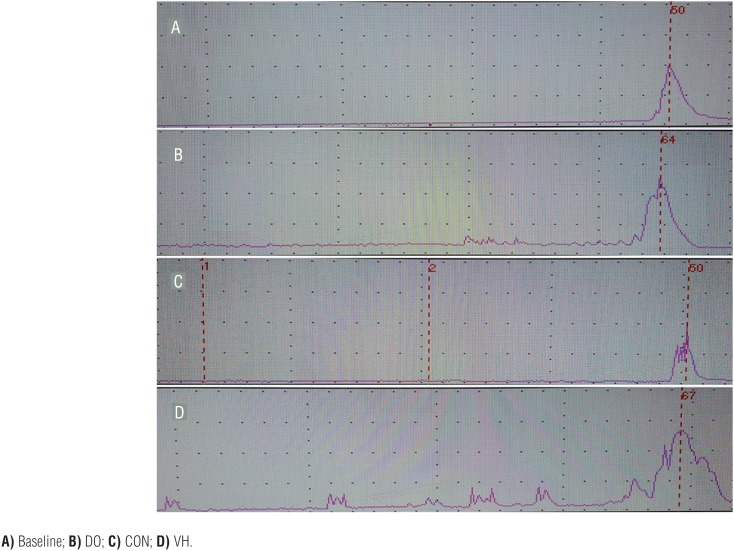
The cystometric investigation of the 4 groups (one voiding contraction is observed and the number indicates peak voiding pressure). **A)** Baseline; **B)** DO; **C)** CON; **D)** VH.

Cystometric investigation was performed in the 4 groups. In the CON group, the bladder filling status was at a low pressure during urine storage, and the detrusor contraction pressure increased significantly during urination and returned to normal status after urination (DO rate=0%, Figure-3C), while the VH group had obvious non-voiding contractions before the onset of voiding contraction and thus was defined as having DO (DO rate=100%, Figure-3D). The peak voiding pressure of the VH group is higher than the CON group (62.5±5.4cm.H_2_O versus 50.3±3.7cm.H_2_O, P=0.024, Table-1).

**Table 1:** Comparison of cystometric investigation between CON and VH group (Mean±SD).

Groups	BC (mL)	P	VP (cm.H_2_O)	P
Baseline	0.28±0.024	0.217^a^	47.8±3.3	
DO	0.79±0.16		67.9±12.8	
CON	0.26±0.031		50.3±3.7	0.024^a^
VH	0.31±0.029		62.5±5.4	

a = CON versus VH.

BC = Bladder capacity.

VP = Peak voiding pressure.

**Figure 4:**
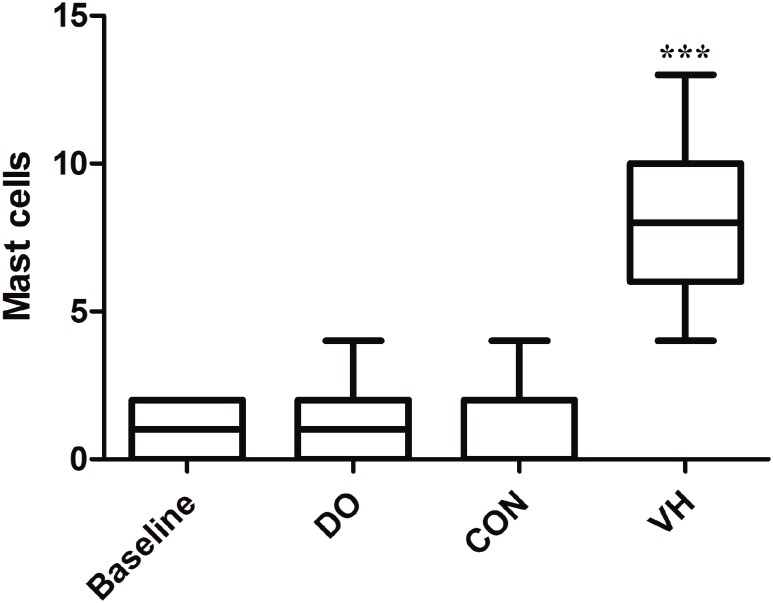
Comparison of MC infiltration of bladder between CON and VH group (1.6±1.4 versus 8.3±6.6, ***P<0.001).

**Figure 5:**
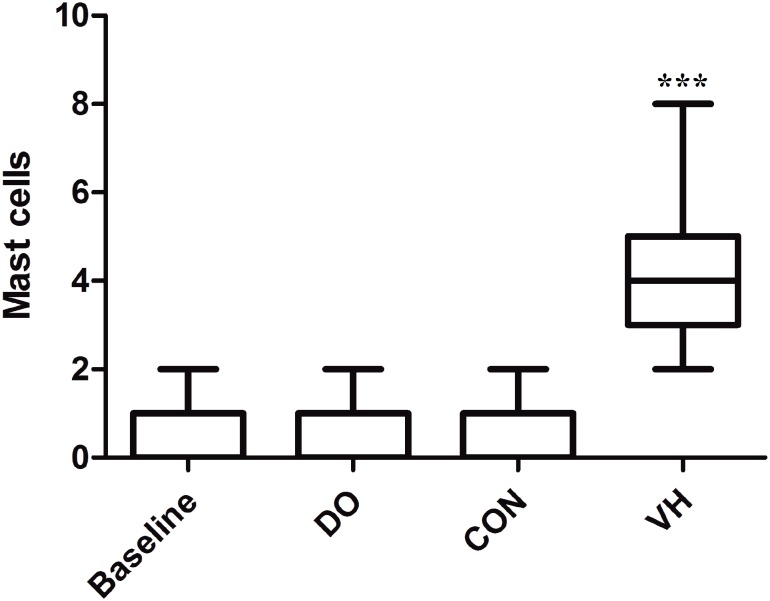
Comparison of MC infiltration of small intestine between CON and VH group (0.67±0.49 versus 4.2±2.8, ***P<0.001).

**Figure 6:**
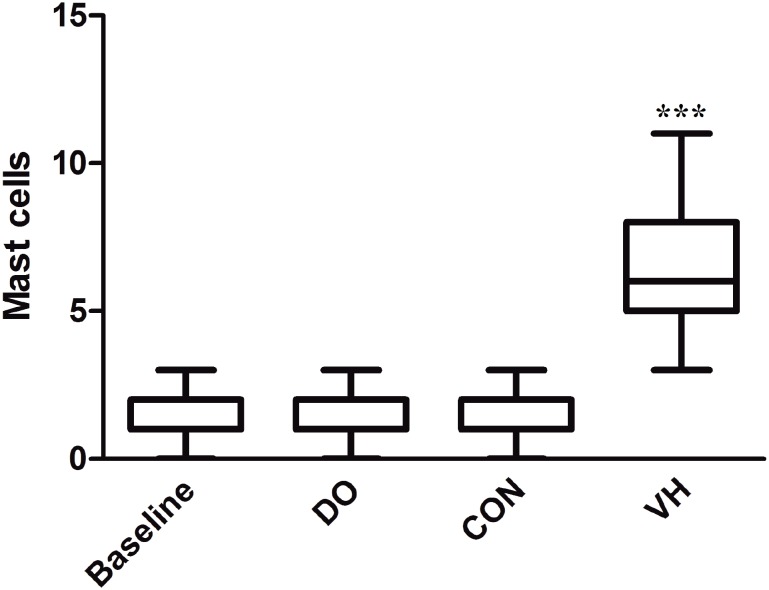
Comparison of MC infiltration of large intestine between CON and VH group (1.6±0.77 versus 6.5±5.0, ***P<0.001).

**Figure 7:**
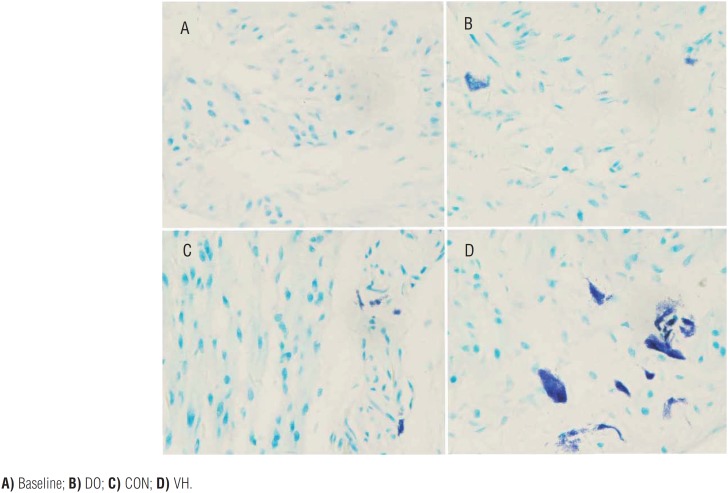
The MC infiltration of bladder in the 4 groups (×400)

**Figure 8:**
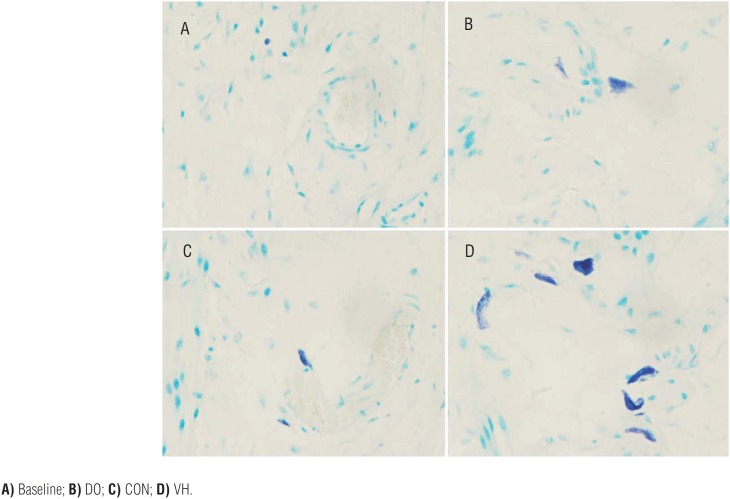
The MC infiltration of small intestine in the 4 groups (×400).

**Figure 9:**
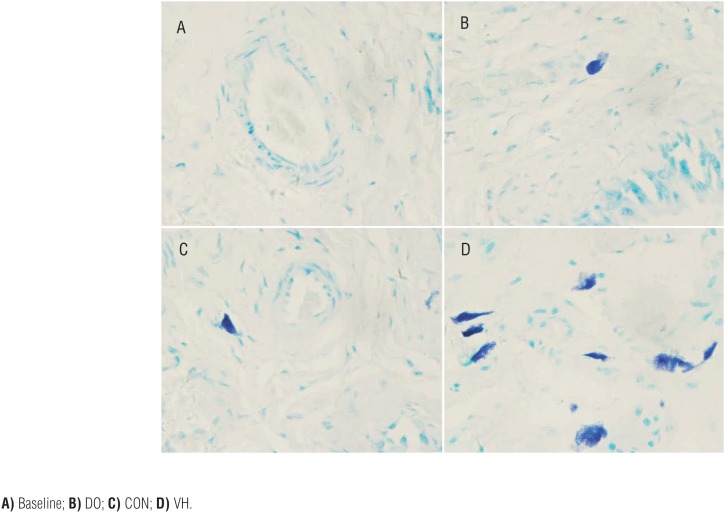
The MC infiltration of large intestine in the 4 groups (×400).

We harvested the bladder and intestine tissue for subsequent histological assessment in the 4 groups. Compared with the CON group, the numbers of MC infiltration were significantly increased in the VH group, including submucosa of bladder (1.6±1.4 versus 8.3±6.6, P<0.001, Figure-4), mucosa lamina propria/mesentery of small intestine (0.67±0.49 versus 4.2±2.8, P<0.001, Figure-5), and mucosa lamina propria/mesentery of large intestine (1.6±0.77 versus 6.5±5.0, P<0.001, Figure-6). Furthermore, more MC activation as well as degranulation showed the pathological changes in the submucosa of bladder and mucosa lamina propria/mesentery of intestine in the VH rats (Figures 7-9).

## DISCUSSION

We established a model of DO in VH rats. Our results confirmed that the VH rats presented altered voiding patterns, and non-voiding contractions occurring prior to voiding were observed in the VH rats. Following voiding, no involuntary detrusor contraction was recorded but the involuntary detrusor contractions developed slowly with increasing amplitude until the next voiding occurred. Interestingly, the DO pattern observed in our study was characterized by an increased involuntary detrusor contractions during the filling phase, which was in agreement with findings in patients showing DO associated with an increase in voiding pressure (20). Therefore, the DO model induced by VH established in our study was consistent with the clinical characteristics of DO patients.

VH has emerged as a key hypothesis in explaining the painful symptoms in IBS and has been proposed as a “biological hallmark” for the condition (21). Moreover, the functional connection between the bladder and bowel, evidenced in studies of the neural crosstalk between these two organs, suggests that they may share some common underlying dysfunction (11, 15). Thus VH, even including MC, may play an important role in the pathophysiological mechanism of DO.

The urinary bladder serves as reservoir that alternates between urine storage and efficient urine expulsion at a convenient moment (22). Normal bladder sensation is the basis to achieve urine storage and urination processes, all bladder neural reflexes and excitement transfers come from the transfer of bladder sensation. More evidence has shown that DO might be a cause of sensory nerve-mediated hypersensitivity or hyperactivity in addition to myogenic excitability (23). There a close anatomic relationship between MC and bladder neuron, which has been demonstrated by electron microscopy (24). MC is found within nerves and intramural ganglia and is in close contact with individual nerve fibers. Close association between MC, nerves, and vessels is common, and ultrastructural evidence suggests that bidirectional communication occurs between MC and nerve fiber. These structures may participate in axon reflexes that regulate detrusor muscle function and cause bladder hyperreactivity (25).

The previous studies revealed that chronic inflammation, such as MC infiltration, was observed in patients with DO (26-29). MC is long-lived, tissue-resident cell that is enriched at boundaries of the body, where it may be especially numerous in the gastrointestinal and genitourinary tracts, adjacent to blood or lymphatic vessels, and near or within peripheral nerves (30). MC mediators are granule-stored, presynthesized molecules or are synthesized de novo (31). MC may be activated by a number of mechanisms within the urinary bladder. Evidence has shown that the urothelium could release a number of neuropeptides (eg, nerve growth factor [NGF]) and neurotransmitters that may activate submucosal afferent nerve and MC (32, 33). These changes in urothelial permeability, urothelial activation, sensory nerve stimulation, and MC activation are complex and highly interrelated with multiple and simultaneous positive and negative feedback loops.

Recent study has shown that MC involved in the inflammatory response to the pathogenesis of OAB, which was frequently associated with DO (29). One pathogeneses, which have been proposed for DO, is the interaction of MC with nerve cell to produce neurogenic inflammation. Patients with DO have been found to have increased urinary NGF level (27, 28). NGF might act as a mediator for bidirectional communication between muscle or urothelium and nerve fibers, including primary sensory afferents (23). The level of NGF in urine could cause DO through some pathways, and it was suggested that MC, the NGF producer, was involved in the pathway of the pathogenesis of DO (29).

In this study, DO model is successfully established in the VH rats, and the common underlying dysfunction between the bladder and bowel is evidenced. Importantly, it is indicated that MC may play an important role in DO. However, our study has several weaknesses. Although our model demonstrated the association between DO and VH, the systematic pathophysiology of DO induced by VH remains unclear. Secondly, our study in animal indicated abnormality of MC infiltration in DO, but the underlying mechanisms can not be confirmed in the absence of further clinical studies to clarify the details.

## CONCLUSIONS

In conclusion, it is indicated that DO model can be established in VH rats. The MC infiltration may play an important role in DO induced by VH, and may be helpful to understand the mechanisms of DO in VH patients.
